# Depression and Incident Hypertension: The Strong Heart Family Study

**DOI:** 10.5888/pcd22.240230

**Published:** 2025-01-16

**Authors:** Serena Santoni, Mary A. Kernic, Kimberly Malloy, Tauqeer Ali, Ying Zhang, Shelley A. Cole, Amanda M. Fretts

**Affiliations:** 1 Institute for Health Metrics and Evaluation, Department of Global Health, University of Washington, Seattle; 2 UW Center on Intimate Partner Violence Research, Policy and Practice, Department of Epidemiology, University of Washington School of Public Health, Seattle; 3 Department of Biostatisitics and Epidemiology, Hudson College of Public Health, Oklahoma City; 4 Department of Biostatistics and Epidemiology, Center for American Indian Health Research, Hudson College of Public Health, The University of Oklahoma Health Sciences Center, Oklahoma City; 5 Center for American Indian Health Research, the University of Oklahoma Health Sciences, Oklahoma City; 6 Population Health, Texas Biomedical Research Institute, San Antonio; 7 Department of Epidemiology, University of Washington, Seattle

## Abstract

**Introduction:**

Compared with White Americans, American Indian adults have disproportionately high depression rates. Previous studies in non-American Indian populations report depression as common among people with uncontrolled hypertension, potentially interfering with blood pressure control. Few studies have examined the association of depressive symptoms with hypertension development among American Indians despite that population’s high burden of depression and hypertension. We examined the association of depressive symptoms with incident hypertension in a large cohort of American Indians.

**Methods:**

We studied 1,408 American Indian participants in the Strong Heart Family Study, a longitudinal, ongoing, epidemiologic study assessing cardiovascular disease and its risk factors among American Indian populations. Depressive symptoms were assessed by using the Center for Epidemiological Studies-Depression (CES-D) scale, 2001–2003. At each study examination in 2001–2003 and 2007–2009, blood pressure was measured 3 times. The average of the last 2 measurements taken at baseline and follow-up examinations was used for analyses. Incident hypertension was defined as systolic blood pressure ≥140 mm Hg, diastolic blood pressure of ≥90 mm Hg, or use of hypertension medications at follow-up. To account for within-family correlation, we used generalized estimating equations to examine the association of depressive symptoms with incident hypertension.

**Results:**

During follow-up, 257 participants developed hypertension. Participants with symptoms consistent with depression (CES-D ≥16) at baseline had 54% higher odds of developing hypertension during follow-up (OR = 1.54; 95% CI, 1.06–2.23) compared with those without depression (CES-D <16) at baseline after adjustment for other risk factors.

**Conclusion:**

These data suggest that participants who experienced symptoms consistent with depression were at increased odds of incident hypertension.

**Summary**

**What is already known on this topic?**

Compared with White Americans, American Indian adults have disproportionately high rates of depression. Previous studies in non-American Indian populations report that depression is common in people with uncontrolled hypertension and may interfere with blood pressure control.

**What is added by this report?**

After adjustment, study participants with depressive symptoms at baseline had 54% higher odds of developing hypertension during the follow-up compared with those without depressive symptoms at baseline.

**What are the implications for public health practice?**

Mental health is a key determinant of cardiovascular disease risk, suggesting the need for mental health outreach programs focusing on depression prevention to mitigate downstream effects on hypertension.

## Introduction

Hypertension and depression are highly prevalent in the US (1,2). However, compared with White American adults, American Indian adults have disproportionately higher rates of cardiovascular risk factors and mental health issues, including hypertension and depression. In 2019, more than 18% (n = 260,000) of surveyed American Indians/Alaska Natives (AI/AN) aged 18 years or older experienced mental illness during the past year (3). Relatedly, in 2018, AI/AN adults were 50% more likely to be diagnosed with coronary heart disease than White adults. In that same year, AI/AN adults were 10% more likely than White adults to have high blood pressure (4). In the Strong Heart Family Study (SHFS), the largest cohort study of cardiometabolic health among American Indians in the US, nearly 50% of participants reported depressive symptoms (5) at the baseline study examination. Of these participants, approximately 30% experienced moderate to severe symptoms of depression.

Previous research has shown that depression is associated with hypertension, and a meta-analysis of prospective cohort studies report depression as an independent risk factor for hypertension (6). Previous studies have also shown that depression is common in people with uncontrolled hypertension and may interfere in blood pressure control (7). To our knowledge, few studies have examined the association of depression with development of hypertension among American Indians, despite the high burden of hypertension and depression in this population.

The purpose of our analysis was to examine the relationship of depressive symptoms with incident hypertension in a large cohort of American Indians. As a secondary exploratory analysis, we also examined whether self-reported experiences with social support affects this relationship in a subset of participants. We hypothesized that participants who reported a greater number of depressive symptoms were more likely to develop hypertension than participants who reported fewer depressive symptoms. Additionally, we hypothesized that the magnitude of the association of depressive symptoms with hypertension would be higher among participants who reported low levels of social support.

## Methods

### Setting and study population

SHFS is a family-based longitudinal study of cardiovascular disease (CVD) and its risk factors in 12 American Indian communities in Arizona, North Dakota, South Dakota, and Oklahoma. The study was designed to better understand genes that contribute to risk of CVD among American Indians (8). The study comprised 2,756 American Indian people aged 14 to 93 years from 92 multigenerational families. The cohort included 409 middle-aged or older participants (14.8%) from the original population-based Strong Heart Study and 3,145 of their spouses, offspring, offspring spouses, and grandchildren. For a family to be eligible to participate in SHFS, a participant of the original Strong Heart Study must have had a minimum of 4 full or half siblings and a total of 12 or more living offspring from the second generation who were aged 18 years or older. Smaller families were not eligible for participation. SHFS participants completed 2 examinations over an 8-year period: a baseline examination in 2001–2003 and a follow-up examination in 2006–2009. Surveillence for morbidity and mortality is ongoing. Each study examination included a personal interview, physical examination, medication review, and laboratory work-up. Data collection procedures have been described in detail in previous publications and are summarized and are summarized by North and colleagues and by Lee and colleagues (8,9). SHFS was approved by the institutional review board of each affected Indian Health Service, and written informed consent was obtained from study participants at each study examination.

Of the 2,756 participants who completed the baseline examination, we excluded SHFS participants who were pregnant (n = 3), because pregnancy may influence the risk of symptoms consistent with depression. Also, we excluded those who had prevalent hypertension (ie, use of antihypertensive medications, diuretics, or beta blockers; SBP ≥140 mm Hg; or DBP ≥90 mm Hg) (n = 713), did not complete the depression assessment (n = 195), or reported taking antidepressant or antipsychotic medications at baseline (n = 48). We excluded participants who reported taking antidepressant or antipsychotic medications at baseline because this may influence the risk of depressive symptoms, potentially clouding study results, and could attenuate the relationship between symptoms consistent with depression and incident hypertension toward the null. We also excluded participants who did not complete the follow-up exam (n = 389). Our total analytic sample comprised 1,408 participants.

### Data collection

**Assessment of depressive symptoms**. The exposure of interest for our study was experiencing symptoms consistent with clinical depression (yes/no). We used the 20-item Center for Epidemiologic Studies Depression (CES-D) scale to assess depressive symptoms at baseline. The CES-D scale is a valid and reliable instrument used to assess depressive symptoms experienced during the past week (10). Symptoms assessed include, for example, feelings of guilt and hopelessness, feeling blue, experiencing insomnia, and the inability to focus. For each question, response options were captured by using a 4-point Likert scale ranging from 0 (none of the time/rarely) to 3 (most of the time). Responses to individual questions were summed after reverse coding of positively framed items per established CES-D guidelines (total possible score range: 0–60). A higher CES-D score is consistent with greater depressive symptomology, and scores of 16 or higher are consistent with diagnoses of major depressive disorder. As in previous SHFS analyses, CES-D scores were categorized as consistent with depression (CES-D ≥16) versus not consistent with depression (CES-D <16) (11).

**Assessment of hypertension**. Our primary outcome of interest was incident hypertension. At each study examination, blood pressure was measured 3 times on the right arm with a standard mercury sphygmomanometer after 5 minutes rest with the participant seated (12). The average of the last 2 measurements taken at both the baseline and follow-up examinations were used for these analyses. Incident hypertension was defined as SBP ≥140 mm Hg, DBP ≥90 mm Hg, or use of hypertension medications at follow-up.

**Measurement of covariates**. Detailed information on important confounding variables including demographic characteristics, diet and physical activity, and other CVD risk factors (eg, smoking status, prevalent diabetes) were collected at the baseline examination (2001–2003) by standardized interviews.

Past-year diet was assessed by using a Block 119-item food frequency questionnaire with an ethnic foods supplement (13). Diet quality was classified by using the Alternative Healthy Eating Index (AHEI) (14). Physical activity was captured using Accusplit AE120 pedometer (8). Average steps per day for each participant were estimated as the mean steps per day across the 3 to 7 days that the pedometer was worn.

Social support was categorized dichotomously based on participants’ response to the question: “Can you count on anyone to provide you with emotional support (talking over problems or helping you make a difficult decision)?” Only a subset of the study sample (n = 332) completed the social support assessment.

Anthropometric measures were obtained with the participant wearing light clothing and no shoes. Bodyweight was measured with a Tanita BWB-800 5 adult digital scale. Height was measured with a vertical-mounted ruler. Body mass index (BMI) was calculated as body weight divided by height-squared (8,9).

Blood samples were collected after a 12-hour overnight fast and were stored at −70 °C. Plasma glucose, LDL cholesterol, and HDL cholesterol were measured by enzymatic methods (9). Diabetes was defined based on 2003 ADA criteria (15), including use of insulin or oral antidiabetic medication or a fasting plasma glucose level greater than or equal to 126 mg/dL.

### Statistical analyses

Two sequential generalized estimating equation (GEE) models were run to assess the association of symptoms consistent with depression with incident hypertension. GEE was used to address potential familial correlation between participants within the data and were run with the assumption of an independent working correlation and specification of robust SEs. In total, 86 family clusters were included in the analysis with a mean of 16 participants per family cluster (range: 1–57 participants per family cluster). Model 1 (minimally adjusted model) adjusted for age (years), sex (male or female), and study site (Arizona, Oklahoma, North Dakota, South Dakota). Model 2 (primary model) further adjusted for CVD risk factors selected a priori based on potential associations with depression and blood pressure, including LDL cholesterol (mg/dL), HDL cholesterol (mg/dL), prevalent diabetes status (yes/no), smoking status (never/former/current), BMI, AHEI (score), and physical activity (steps per day).

In the exploratory analyses, we examined potential interaction of depression and social support with incidence of hypertension by inclusion of an interaction term (depression*social support) in the primary model and using a Wald test for significance. We used complete case analysis to conduct analyses. All statistical analyses were performed in R software (R Foundation).

## Results

The median age of study participants was 33.5 years (range, 14.1 y–86.0 y) and 36.5% of the analytic cohort identified as male. Baseline characteristics of study participants were assessed according to CES-D (<16 or ≥16) (Table 1). At baseline, 27.3% (n = 385) of study participants reported symptoms consistent with depression (ie, CES-D score, ≥16). Participants who reported baseline symptoms consistent with depression were more likely to be female (74.0% vs 59.5%), were slightly younger (32.6 y vs 35.4 y), had less education (11.5 y vs 12.4 y), had a higher BMI (31.2 vs 29.9), and reported less physical activity (5,640 steps per day versus 6,690 steps per day) compared with participants with a CES-D score of <16. There was no difference in diet quality based on CES-D score.

**Table 1:** Characteristics of Study Participants^a ^(N = 1,408), by Baseline Depressive Symptoms, CES-D Scale^b^

Characteristic	CES-D score not consistent with depression^c^ (N = 1,023)	CES-D score consistent with depression^d^ (N = 385)	Total sample (N = 1,408)
**Sex, n (%)**			
Male	414 (40.5)	100 (26.0)	514 (36.5)
Female	609 (59.5)	285 (74.0)	894 (63.5)
**Age, y, mean (SD)**	35.4 (14.7)	32.6 (12.8)	34.6 (14.3)
**Education, y, mean (SD)**	12.4 (2.19)	11.5 (2.17)	12.2 (2.23)
**BMI (kg/m^2^), mean (SD)**	29.9 (6.88)	31.2 (8.28)	30.2 (7.31)
**Diabetes, n (%)**			
Yes	89 (8.7)	43 (11.2)	132 (9.4)
No	929 (90.8)	339 (88.1)	1268 (90.1)
**Current smoker, n (%)**			
Yes	375 (36.7)	182 (47.3)	557 (39.6)
No	647 (63.2)	202 (52.5)	849 (60.3)
**AHEI, mean (SD)**	43.4 (9.06)	43.2 (8.76)	43.3 (8.98)
**Physical activity (continuous, steps per day), mean (SD)**	6,690 (3,980)	5,640 (3,510)	6,400 (3,880)

Abbreviations: AHEI, Alternative Healthy Eating Index; BMI, body mass index; CES-D, Center for Epidemiologic Studies Depression scale.

a The Strong Heart Family Study (8).

b Symptoms are scored per established CES-D guidelines (total possible score range: 0–60). A higher CES-D score is consistent with greater depressive symptomology, and scores of 16 or higher are consistent with diagnoses of major depressive disorder.

c CES-D <16. Missingness removed; therefore, not all columns total 100%.

d CES-D ≥16. Missingness removed; therefore, not all columns total 100%.

During the follow-up period, 257 participants developed hypertension (Table 2). Participants who developed hypertension were more likely to be older (42.0 years old vs 33.0 years old), male (44.7% vs 34.7%), and have prevalent diabetes at baseline (21.8% vs 6.6%), reported fewer steps per day (5,730 steps per day vs 6,540 steps per day), and had higher BMI (32.4 vs 29.8) than participants who did not develop hypertension during follow-up. Additionally, participants who developed hypertension were more likely to smoke (43.2% vs 38.7%) than participants who did not develop hypertension. We observed no differences in education or diet quality based on hypertension status.

**Table 2:** Demographic and Health Characteristics by Hypertension^a^ Status at Follow-Up, Participants^b^ (N = 1,408)

Characteristic	No hypertension (n = 1,151)	Hypertension (n =257)	Total (n =1,408)
**Depression, n (%)**			
Not consistent with depression (CES-D <16)^c^	844 (73.3)	179 (69.6)	1023 (72.7)
Consistent with depression (CES-D ≥16)^b^	307 (26.7)	78 (30.4)	385 (27.3)
**Sex, n (%)**			
Male	399 (34.7)	115 (44.7)	514 (36.5)
Female	752 (65.3)	142 (55.3)	894 (63.5)
**Age, y, mean (SD)**	33.0 (13.6)	42.0 (14.9)	34.6 (14.3)
**Education, y, mean (SD)**	12.1 (2.23)	12.4 (2.22)	12.2 (2.23)
**BMI (kg/m^2^), mean (SD)**	29.8 (7.15)	32.4 (7.63)	30.2 (7.31)
**Diabetes, n (%)**			
Yes	76 (6.6)	56 (21.8)	132 (9.4)
No	1068 (92.8)	200 (77.8)	1268 (90.1)
**Current smoker, n (%)**			
Yes	446 (38.7)	111 (43.2)	557 (39.6)
No	703 (61.1)	146 (56.8)	849 (60.3)
**AHEI, mean (SD)**	43.1 (9.09)	44.5 (8.40)	43.3 (8.98)
**Physical activity (continuous, steps per day), mean (SD)**	6,540 (3,870)	5,730 (3,870)	6,400 (3,880)

Abbreviations: AHEI, Alternative Healthy Eating Index; BMI, body mass index; CES-D, Center for Epidemiologic Studies Depression Scale.

a Hypertension defined as systolic blood pressure of ≥140 mm Hg, diastolic blood pressure of ≥90 mm Hg, or use of hypertension medications at follow-up.

b The Strong Heart Family Study (8).

c Symptoms are scored per established CES-D guidelines (total possible score range: 0–60). A higher CES-D score is consistent with greater depressive symptomology, and scores of 16 or higher are consistent with diagnoses of major depressive disorder.

In multivariable GEE analyses, participants with baseline symptoms consistent with depression (CES-D ≥16) had 54% higher odds of developing hypertension during the 3- to 8-year follow-up (OR = 1.54; 95% CI, 1.06–2.23) compared with those with baseline symptoms not consistent with depression (CES-D <16) after adjustment for age, demographic, behavioral, and dietary factors (Table 3).

**Table 3:** Odds of Incident Hypertension^a^, by Depressive Symptom Exposure, All Study Centers, Participants^b^ (N = 1,408)

Variable	Model 1^c^, OR (95% CI) (n = 1,408)	Model 2^d^, OR (95% CI) (n = 1,168)
**Symptom category**		
Not consistent with depression (CES-D <16^e^)	1 [Reference]	1 [Reference]
Consistent with depression (CES-D ≥16^e^)	1.54 (1.12–2.11)	1.54 (1.06 – 2.23)

Abbreviations: BMI, body mass index; CES-D, Center for Epidemiologic Studies Depression scale; OR, odds ratio.

a Hypertension defined as systolic blood pressure of ≥140 mm Hg, diastolic blood pressure of ≥90 mm Hg, or use of hypertension medications at follow-up.

b The Strong Heart Family Study (8).

c Model 1: Adjusted only for age, sex, education, study center.

d Model 2: Further adjusted for HDL cholesterol, LDL cholesterol, smoking status, BMI, diabetes status, physical activity, diet index.

e Symptoms are scored per established CES-D guidelines (total possible score range: 0–60). A higher CES-D score is consistent with greater depressive symptomology, and scores of 16 or higher are consistent with diagnoses of major depressive disorder.

We found no significant interaction of baseline depression status with social support on odds of hypertension in a model adjusted for age, sex, education, study center, baseline blood pressure measurement, HDL cholesterol, LDL cholesterol, smoking status, BMI, diabetes status, physical activity, diet index, sex, study site, and prevalent diabetes (*P* = .35).

## Discussion

In our large cohort study of American Indian adults, participants who reported symptoms consistent with depression at baseline were more likely to develop hypertension when compared with participants who did not report symptoms consistent with depression. This finding supports the hypothesis that depression is associated with increased odds of incident hypertension.

Our findings are consistent with the findings from various prospective studies (17–19) and 1 meta-analysis of 9 (6) studies in non-American Indian populations that show a positive association between depressive symptoms and hypertension. Although these studies used a wide variety of instruments to assess depressive symptoms (eg, Depression Anxiety Stress Scale (DASS-21) [19], National Epidemiologic Survey on Alcohol and Related Conditions [17], 30-item General Health Questionnaire Depression subscale [18]), these findings highlight a positive association of depressive symptoms with hypertension in diverse populations across a wide range of ages and geographic contexts.

Our findings are discordant with several studies that reported no (or inverse) associations betweem depressive symptoms and incident hypertension (20,21). Differences in study populations according to age and geography may account for contradictory findings across studies (22). For instance, although it has not been extensively studied, the etiology of depressive symptoms possibly may be different in old versus young populations (23). Additionally, access to quality health care (including mental health services) differs according to area of residence (eg, urban vs rural, US vs non-US, American Indian reservation vs nonreservation). Finally, the lived experiences of American Indians are different than those of non-American Indians, including the impact of multigenerational historical trauma and structural racism on mental and physical health.

To our knowledge, no studies to date have examined the relationship of depression with the development of hypertension in American Indians. In 1 cross-sectional study among 500 older AI/AN adults who resided in urban areas in the Pacific Northwest, clinic patients with prevalent hypertension were more likely to have depression than patients without hypertension (24). However, this cross-sectional study was unable to infer whether depression increased odds of hypertension or vice versa. Our work complements findings from the SHFS that reported that participants with severe depressive symptoms (ie, CES-D ≥16) have a 71% higher odds of developing CVD compared with participants who did not report symptoms consistent with depression (OR = 1.71; 95% CI, 1.01–2.91) (25).

Several studies of Indigenous populations point to high levels of depression and CVD risk factors and diseases (26–28). The high burden of depressive symptoms among American Indians may be due at least in part to generations of oppression and historical trauma, including forced migration to reservations, abuse and neglect of American Indian young people at government-operated boarding schools, and near-eradication of many tribal languages, spiritual practices, and cultures (29). Historical trauma and present-day socioeconomic factors may also affect the availability, accessibility, and use of mental and physical health services by American Indian communities. Because of historically poor interactions with the US government, present-day American Indian communities may have lost trust in many institutional sources, including some health care settings (30). Additionally, given the historical relationship between American Indian and US government authorities, many American Indians may prefer to seek care from American Indian mental and physical health care providers, who are scarce (31). The effects of a long history of oppression, colonization, and genocide have had lasting effects on the health of American Indians, which may explain in part the high rates of depression and CVD risk in many of their communities.

The mechanism by which depressive symptoms may influence odds of hypertension is multifaceted and includes stress, inflammation, and neurotransmission processes. Studies have shown that depression can increase the body’s sympathetic tone and cortisol, which increase systemic inflammation, and lead to many cardiometabolic risk factors, including hypertension (32). Established research has linked dopamine to depression because this neurotransmitter plays a vital role in a person’s ability to experience pleasure. Specifically, a dopamine deficit has been linked to anhedonia, the core feature of major depressive disorder (33). Recent studies have shown that lack of dopamine at key brain sites can increase blood pressure (7). Depression may also increase risk of key cardiovascular risk factors, including physical inactivity, obesity, poor dietary practices, and low social support (34).

We did not observe a significant effect of the interaction of social support with depression status on odds of incident hypertension. This may be due in part to our incomplete measurement of social support — we had only 1 question on social support — or the limited power of this measure, because this question was only asked of a subset of SHFS participants (n = 332). Future studies are needed that include a comprehensive measure of social support and community and cultural engagement to better assess whether social support may mitigate the risk of symptoms consistent with depression on incident hypertension.

Our study has many strengths. To our knowledge, ours is the first to examine the association of depressive symptoms with incident hypertension in a well-characterized multitribal cohort of American Indians with detailed measures of depressive symptoms, hypertension, and key covariates. However, our study is not without limitations. Although the CES-D scale has been shown to be a valid and reliable measure of depressive symptoms in noninstitutionalized diverse populations (10), it is susceptible to social desirability bias. Residual confounding by unmeasured or poorly measured factors is possible. Finally, although these results are generalizable to American Indians from large families who reside in primarily rural communities in the Great Plains, Midwest, and Southwestern regions of the US, it is unclear whether findings are generalizable to other populations.

In conclusion, in this large study of American Indian adults, symptoms consistent with depression were found to be positively associated with incident hypertension. The study adds to a growing body of evidence identifying mental health as a key determinant of CVD risk and suggests the need for mental health outreach programs that focus on prevention of depression to mitigate downstream effects on hypertension.
